# Local *GHR* roles in regulation of mitochondrial function through mitochondrial biogenesis during myoblast differentiation

**DOI:** 10.1186/s12964-023-01166-5

**Published:** 2023-06-19

**Authors:** Bowen Hu, Changbin Zhao, Xiangchun Pan, Haohui Wei, Guodong Mo, Mingjian Xian, Wen Luo, Qinghua Nie, Hongmei Li, Xiquan Zhang

**Affiliations:** 1 State Key Laboratory of Livestock and Poultry Breeding, South China Agricultural University, Guangzhou, Guangdong China; 2 State Key Laboratory for Conservation and Utilization of Subtropical Agro-Bioresources and Lingnan Guangdong Laboratory of Agriculture, South China Agricultural University, Guangzhou, Guangdong China; 3 Department of Animal Genetics, Breeding and Reproduction, College of Animal Science, South China Agricultural University, Guangzhou, Guangdong China

**Keywords:** *GHR*, *IGF1*, Mitochondrial biogenesis, Mitochondrial function, Myoblast differentiation

## Abstract

**Background:**

Myoblast differentiation requires metabolic reprogramming driven by increased mitochondrial biogenesis and oxidative phosphorylation. The canonical GH-GHR-IGFs axis in liver exhibits a great complexity in response to somatic growth. However, the underlying mechanism of whether local *GHR* acts as a control valve to regulate mitochondrial function through mitochondrial biogenesis during myoblast differentiation remains unknown.

**Methods:**

We manipulated the *GHR* expression in chicken primary myoblast to investigate its roles in mitochondrial biogenesis and function during myoblast differentiation.

**Results:**

We reported that *GHR* is induced during myoblast differentiation. Local *GHR* promoted mitochondrial biogenesis during myoblast differentiation, as determined by the fluorescence intensity of Mito-Tracker Green staining and MitoTimer reporter system, the expression of mitochondrial biogenesis markers (*PGC1α*, *NRF1*, *TFAM*) and mtDNA encoded gene (*ND1*, *CYTB*, *COX1*, *ATP6*), as well as mtDNA content. Consistently, local *GHR* enhanced mitochondrial function during myoblast differentiation, as determined by the oxygen consumption rate, mitochondrial membrane potential, ATP level and ROS production. We next revealed that the regulation of mitochondrial biogenesis and function by *GHR* depends on *IGF1*. In terms of the underlying mechanism, we demonstrated that *IGF1* regulates mitochondrial biogenesis via PI3K/AKT/CREB pathway. Additionally, *GHR* knockdown repressed myoblast differentiation.

**Conclusions:**

In conclusion, our data corroborate that local *GHR* acts as a control valve to enhance mitochondrial function by promoting mitochondrial biogenesis via IGF1-PI3K/AKT/CREB pathway during myoblast differentiation.

Video Abstract

**Supplementary Information:**

The online version contains supplementary material available at 10.1186/s12964-023-01166-5.

## Introduction

Growth hormone receptor (GHR) belongs to the class I cytokine receptor family, which is an amino acid dimeric receptor contains an extracellular domain (ECD), a single-pass transmembrane domain (IMD) and a cytoplasmic intracellular domain (ICD) [1, 2]. Following the binding of GH to GHR, manifold signal cascades are activated, including Janus kinases (JAKs)/signal transducers and activators of transcription (STATs) [3], mitogen-activated protein kinases (MAPK) [4], phosphoinositide-3-kinase (PI3K)/Protein kinase B (PKB or AKT) [5] and phospholipase C (PLC)/Protein kinase C (PKC) [6]. One of the well documented is the GH-GHR-IGFs axis, in which GH combines with GHR to regulate insulin-like growth factor 1 (IGF1), IGF2, IGF-binding protein 3 (IGFBP3) and acid labile subunit (ALS) through JAK2/STAT5 pathway [7]. As part of the somatotropic hypothalamic-pituitary system, the canonical GH-GHR-IGFs axis in liver exhibits a great complexity in response to somatic growth, including cell proliferation, differentiation, division, and survival.

Skeletal muscle is constructed by a cohort of muscle fibers generated from myoblast [8]. The process of myoblast proliferation and differentiation into myotube, also termed as myogenesis, is an intricate process requiring a precisely controlled regulation that occurs during embryonic development, as well as muscle regeneration and repair [9]. Therefore, myoblasts play a pivotal role in skeletal muscle growth and formation. In mammals, GH promotes C2C12 cell proliferation and inhibits its differentiation through an autocrine manner [10]. In poultry, chicken GH (cGH) does not affect its daily gain, feed conversion rate or muscle growth [11, 12]. However, cGH increases *GHR* expression in vitro to promote the proliferation of satellite cells and inhibit their differentiation [13]. Knockout of *GHRKO* or *IGF1R* in mouse skeletal muscle elicits impaired muscle development and a decrease in the number and size of muscle fibers, which can be attributed to the reduction of myoblast fusion during muscle development [14, 15]. Our previous research revealed that mutations in *GHR* render a decrease in the number and diameter of muscle fibers in 14-embyro-age and 7-week-age sex-linked dwarf (SLD) chicken [16], indicating that local *GHR* may affect the growth and development of skeletal muscle in the embryonic stage.

Cellular adenosine triphosphate (ATP) is mainly generated by mitochondria through oxidative phosphorylation (OXPHOS). During the process of cancer cell proliferation, mitochondria must remain repressive to promote cell proliferation. Even in the presence of oxygen, energy still preferentially obtains from the glycolysis, which is termed as “Warburg effect” [17]. Consequently, raising mitochondrial activity can inhibit myoblast proliferation [18]. On the contrary, myotube is a highly metabolically active cell type, and heavily depends on OXPHOS to provide ATP [18]. Evidence for mitochondrial dysfunction inhibiting myoblast differentiation has been provided by multiple studies with a series of model systems [19–24]. Thus, myoblast differentiation requires metabolic reprogramming, which leads to an increase in OXPHOS and mitochondrial mass through regulating mitochondrial biogenesis [25–30]. Mitochondrial biogenesis is a self-renewal process that requires coordination between mitochondrial DNA (mtDNA) and nuclear DNA (nDNA), including mtDNA transcription and translation, translation of nDNA encoded transcripts, protein import and assembly of the OXPHOS complexes [31]. Our previous review has summarized the versatile relationship between GH-GHR-IGF1 axis, mitochondrial biogenesis and mitochondrial function, and postulated that the effects of GH-GHR-IGFs axis on mitochondrial biogenesis and function might be mostly mediated by IGF1 [32]. However, the roles of local *GHR* in the regulation of mitochondrial biogenesis and function during myoblast differentiation is not clear.

Given this, we manipulated the *GHR* expression in chicken muscle stem cell to investigate its roles in mitochondrial biogenesis and function during myoblast differentiation. We found that local *GHR* acts as a control valve to enhance mitochondrial function by promoting mitochondrial biogenesis via IGF1-PI3K/AKT/CREB during myoblast differentiation. Understanding the precise roles of local *GHR* in myoblast differentiation may provide attractive tools for the development of effective molecular therapies to treat muscle-related diseases, including sarcopenia and muscle atrophy. In the future, this may also pave the new avenues for the development of new strategies targeting mitochondria to promote muscle development for the cultivated meat industry and even improve some muscle developmental defects.

## Materials and methods

### Cell culture

Chicken primary myoblast was isolated from the chicken leg muscle on embryonic 11 day as previous described [33]. Chicken primary myoblast (CPM) was cultured with growth medium (GM) consisting of RPMI-1640 medium (Gibco, USA), 15% fetal bovine serum (FBS) (Gibco, USA), and 0.2% penicillin/streptomycin. After myoblasts achieving 90% cell confluence, the GM was then removed and replaced with differentiation medium (DM) consisting of RPMI-1640 medium without FBS, 2% horse serum and 0.2% penicillin/streptomycin. All cells were cultured at 37 °C in a 5% CO_2_ humidified atmosphere.

### RNA extraction and real-time quantitative PCR

Total RNA was extracted from cells with RNAiso reagent (Takara, Japan) according to the manufacturer’s protocol. The RNA integrity and concentration were determined using 1.5% agarose gel electrophoresis and a Nanodrop 2000c spectrophotometer (Thermo, USA), respectively. cDNA was synthesized using PrimeScript RT reagent Kit (Takara, Japan) for Real-Time quantitative PCR (RT-qPCR). The MonAmp™ ChemoHS qPCR Mix (Monad, China) was utilized for RT-qPCR in a Bio-Rad CFX96 Real-Time Detection instrument (Bio-Rad, USA) according to the manufacturer’s protocol. Relative gene expression was measured by RT-qPCR and nuclear gene *β-actin* was utilized as a control. The primers utilized in RT-qPCR were shown in Table S1 and synthesized by Sangon Biotech (Shanghai, China).

### DNA extraction and analysis of mtDNA copy number

Total nuclear DNA and mtDNA were extracted from cells with a DNA tissue kit (Omega, USA) according to the manufacturer’s protocol. The DNA integrity and concentration were determined using 1.5% agarose gel electrophoresis and a Nanodrop 2000c spectrophotometer (Thermo, USA), respectively. The MonAmp™ ChemoHS qPCR Mix (Monad, China) was utilized for RT-qPCR in a Bio-Rad CFX96 Real-Time Detection instrument (Bio-Rad, USA) according to the manufacturer’s protocol. Relative mtDNA copy number was measured by RT-qPCR performed twice for each reaction using specific primers for mtDNA *ND1* gene and alternate primers for mtDNA *tRNA-Leu* gene (NC_053523.1), a nuclear single-copy gene *β2M* was utilized as a control. The primers utilized in RT-qPCR were shown in Table S1 and synthesized by Sangon Biotech (Shanghai, China).

### RNA interference

The siRNAs used for the knockdown of *GHR*, *IGF1* and *CREB* were synthesized by Guangzhou RiboBio (Guangzhou, China). In our preliminary experiments, we designed three siRNA for each gene and selected the siRNA with the highest interference efficiency. si-*GHR*, si-*IGF1*, si-*CREB* and si-NC were transfected in cells to a final concentration of 150 nM, and cells were analyzed at 48 h after transfection. The sequence of siRNA was shown in Table S2. The inhibition efficiencies were detected by the fluorescence intensity of Cy3 siRNA and RT-qPCR.

### Plasmids construct

Overexpression vectors: *GHR* coding sequence (NCBI Reference Sequence: NM_001001293.2), *IGF1* coding sequence (NCBI Reference Sequence: NM_001004384.3) and *CREB1* coding sequence (NCBI Reference Sequence: NM_ NM_204450.3) were amplified from chicken cDNA and cloned into the pcDNA3.1 vector (Invitrogen, USA). PGC1α promoter reporter plasmids were amplified from chicken cDNA cloned into the pGL3-basic luciferase reporter vector (Promega, USA). p*MitoTimer* was a gift from Zhen Yan (Addgene plasmid, 52,659), and empty vector p*CI-neo* (Promega, USA) was utilized as internal control. All plasmid constructs were confirmed by DNA sequencing.

### Transfection

Cells were plated in culture plates and incubated overnight prior to the transfection experiment. Transfection was performed with the Lipofectamine 3000 reagent (Invitrogen, USA) following the manufacturer’s protocol and nucleic acids were diluted in OPTI-MEM Medium (Gibco, USA). Transfection was performed when myoblasts achieved 90% cell confluence, GM was then removed and replaced with DM after transfection was complete. All cells were analyzed at 48 h after transfection.

### Mito-Tracker Green staining and Hoechst 33,342 staining

Mito-Tracker Green (MTG) staining and Hoechst 33,342 staining was used to label the mitochondria and nuclei in cells, respectively. DMSO (Beyotime, Shanghai, China) was utilized as internal control. Cells were washed twice with PBS and incubated with Mito-Tracker Green (Beyotime, Shanghai, China) for 30 min at 48 h after transfection. Cells were then suspended in PBS and 10 μL of Hoechst 33,342 dye was added (Beyotime, Shanghai, China). After being washed in PBS twice, a fluorescence microscope (TE2000-U; Nikon, Japan) was used to capture five randomly selected fields and analyzed with NIS-Element’s software.

### Western blot analysis

Cellular protein was lysed by radio immune precipitation assay (RIPA) buffer (Beyotime, China) with phenylmethane sulfonyl fluoride (PMSF) protease inhibitor (Beyotime, China) and the homogenate was centrifuged at 12,000 × g for 5 min at 4 ^◦^C. The supernatant was collected and the protein concentration was determined immediately using a BCA protein quantification kit (Beyotime, China). The proteins were separated in 10% SDS-PAGE and transferred onto PVDF membrane, and then probed with antibodies following standard procedures. The antibodies and their dilutions utilized for western blots were as follow: anti-GHR (bs-0654R; Bioss, China; 1:500), anti-PGC1α (bs-1832R; Bioss, China; 1:500), anti-NRF1 (12,482–1-AP; Proteintech, USA; 1:500), anti-TOMM20 (AF1717; Beyotime, China; 1:500), anti-JAK2 (bs-0908R; Bioss, China; 1:1000), anti-p-JAK2 (bsm-52171R; Bioss, China; 1:1000), anti-AKT1 (bs-0115 M; Bioss, China; 1:500), anti-p-AKT1 (66,444–1-Ig; Proteintech, China; 1:500), anti-CREB1 (bs-0035R; Bioss, China; 1:500), anti-p-CREB1 (bs-0036R; Bioss, China; 1:500), anti-β-actin (bs-0061R; Bioss, China; 1:1000), goat anti-rabbit IgG-HRP (bs-0295G; Bioss, China; 1:5000), goat anti-mouse IgG-HRP (bs-0296G; Bioss, China; 1:5000).

### Dual-luciferase reporter assay

For promoter validation, the cells were transfected with a series of the promoter reporter plasmids described above and the TK-Renilla reporter (Promega, USA) was co-transfected as internal control. For interaction assays, the promoter reporter plasmids were co-transfected with the *CREB* overexpression vector in CPM and the TK-Renilla reporter (Promega, USA) was co-transfected as internal control. At 48 h after transfection, the luciferase activities of the cells were measured using the Dual-Glo® Luciferase Assay System (Promega, USA) and Fluorescence/Multi-Detection Microplate Reader (BioTek, USA). The levels of firefly luciferase activity were normalized to Renilla luciferase activity.

### Detection of reactive oxygen species

Reactive oxygen species (ROS) production was measured using an ROS assay kit (Beyotime, China) according to the manufacturer’s protocol. Dichlorofluorescein (DCF) fluorescence was determined using a Fluorescence/Multi-Detection Microplate Reader (BioTek, USA).

### Detection of ATP content

ATP levels were measured using an ATP assay kit (Beyotime, China) according to the manufacturer’s protocol. A Fluorescence/Multi-Detection Microplate Reader (BioTek, USA) was used to determine luminescence level.

### Detection of mitochondrial membrane potential

Mitochondrial membrane potential (ΔΨm) was measured using a JC-1 kit (Beyotime, China) according to the manufacturer’s protocol. The fluorescence was determined using a flow cytometer (BD Biosciences, USA) after the cells were incubated with JC-1 for 20 min at 37 °C; 10 µM rotenone was used as a standard inhibitor of ΔΨm.

### Oxygen consumption

Oxygen consumption rate (OCR) in CPM was measured utilizing a Seahorse XF Cell Mito Stress Test Kit (103,015, Agilent Technologies, USA) and Seahorse Extracellular Flux Analyzer (Agilent) according to the manufacturer’s protocol. Cells were plated on a miniplate 24 h prior to the Seahorse assay. The OCR was monitored upon following sequential injections of oligomycin (1 µM), FCCP (1 µM), and a rotenone/antimycin A mixture (1.5 µM).

### Cell counting kit-8 assays

Cells were seeded in 96-well plates and cultured in growth medium. After transfection, the proliferation of the cell culture was monitored at 12, 24, 36 and 48 h using a Cell Counting Kit-8 (CCK-8) kit (Beyotime, China) according to the manufacturer’s protocol. The absorbance at 450 nm was determined using a Fluorescence/Multi-Detection Microplate Reader (BioTek, USA). The data were acquired by averaging the results from six independent repeats.

### 5-ethynyl-20-deoxyuridine assays

Cells were seeded in 24-well plates and cultured to 50% density for transfection. Cells were fixed and stained with a 5-ethynyl-20-deoxyuridine (EdU) imaging kit (RiboBio, China) according to the manufacturer’s protocol. A fluorescence microscope (DMi8; Leica, German) was used to capture three randomly selected fields to visualize the number of EdU stained cells.

### Immunofluorescence

The immunofluorescence was performed using anti-MyHC (B103; DSHB, USA; 1:50). After transfection for 48 h, cells were fixed in 4% formaldehyde for 20 min then washed three times with PBS for 5 min. Subsequently, the cells were permeabilized by adding 0.1% Triton X-100 for 15 min and blocked with goat serum for 30 min. After overnight incubation with anti-MyHC at 4 °C, the Dylight 594-conjugated AffiniPure Goat Anti-Mouse IgG (H + L) (BS10027; Bioworld, USA; 1:100) was added and the cells were incubated in dark for 1 h. The cell nuclei were stained with DAPI (Beyotime, China). Results were visualized on a fluorescence microscope (DMi8; Leica, German) and measured by using ImageJ software (National Institutes of Health). Myotube area was calculated as the percentage of the total image area covered by myotubes.

### Statistical analysis

All the experiments were performed at least three times. The graphical representation was performed in GraphPad Prism v9.0 software (GraphPad Software, USA). The error bar was presented as means ± standard error of the mean (S.E.M.). The statistical analyze was performed using two-sided Student’s *t*-test, and we considered *p* < 0.05 to be statistically significant, *p < 0.05, **p < 0.01.

## Results

### High* GHR* expression during myoblast differentiation

To understand whether local *GHR* affects the growth and development of skeletal muscle during embryonic stage, we first investigated the *GHR* expression profile in the myoblast proliferation and differentiation phases. *GHR* expression increased gradually during myoblast differentiation, up to tenfold in comparison to proliferating cells (Fig. S1a). In vivo, *GHR* is highly expressed during embryo age 10 (E10)-E14, but is relatively low after E18 in leg muscle [34]. These results suggest that *GHR* mainly play its roles during myoblast differentiation.

Myoblasts demand two stages to form the multinucleated myotubes in vitro [14, 15]. First, two myoblasts fuse to form a nascent myotube at 24 h after induction of differentiation. During the next phase (24–48 h), additional myoblasts fuse to this nascent myotube to produce a fully differentiated myotube. Therefore, we overexpressed or interfered with related genes during the myoblast differentiation phase (Fig. S1b). To confirm that plasmid or siRNA was successfully transfected into cells during myoblast differentiation, we utilized pcDNA3.1-EGFP or siNC-Cy3 to determine the transfection efficiency (Fig. S1c, d).

### Local *GHR* promotes mitochondrial biogenesis

**Fig. 1:**
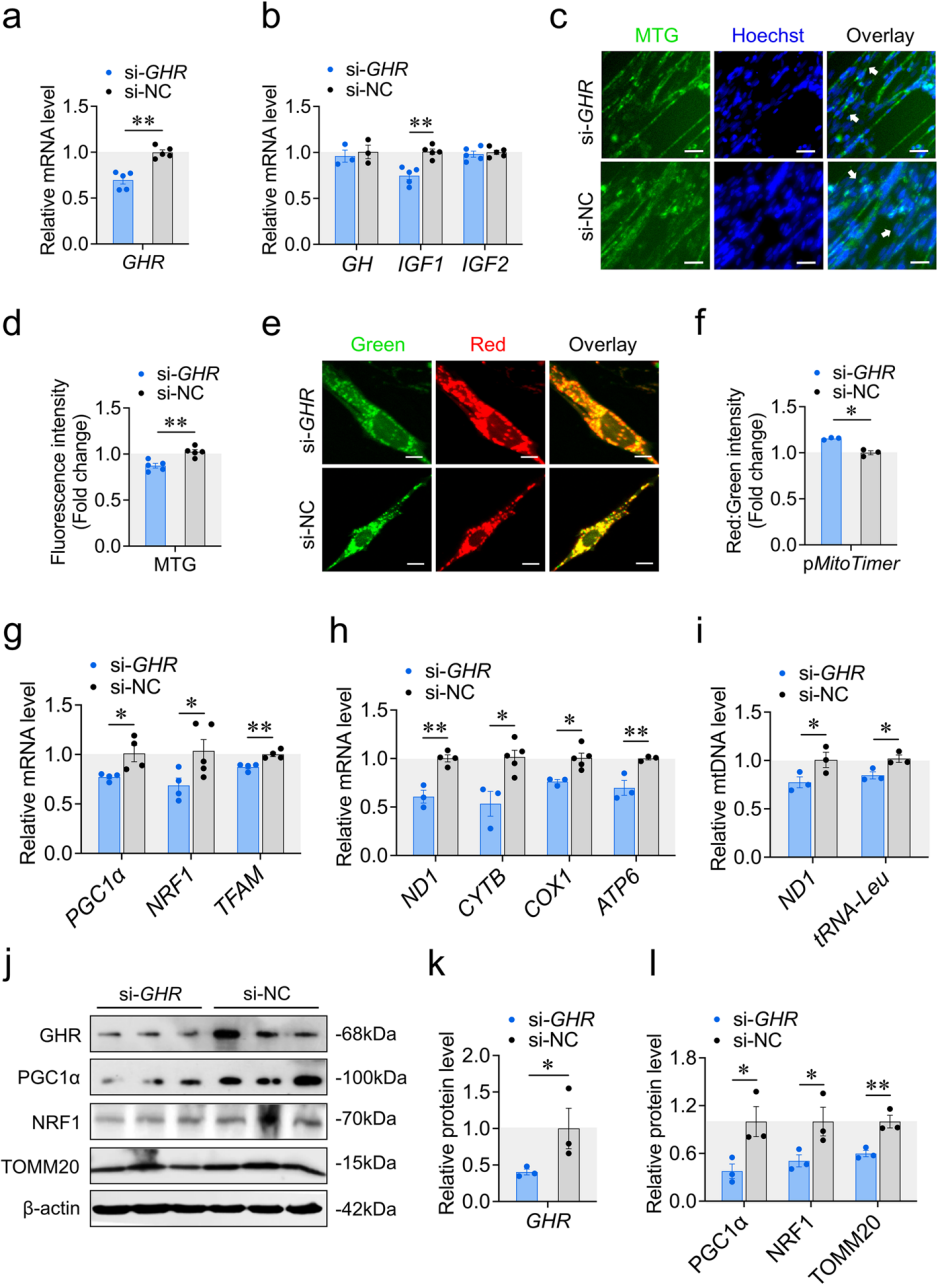
*GHR* knockdown inhibits mitochondrial biogenesis. **a** Knockdown efficiency was measured by RT-qPCR at 48 h after transfection with si-*GHR* and si-NC. **b** The expression of genes involved in the GH-GHR-IGFs signaling pathway was measured by RT-qPCR at 48 h after transfection with si-*GHR* and si-NC. **c** and **d** MTG staining of CPM was measured at 48 h after transfection with si-*GHR* and si-NC. White arrow labeled elongated myoblasts. Scaler bar, 25 μm. **e** and **f** Confocal images were observed at 48 h after co-transfection with p*MitoTimer* + si-*GHR* and p*MitoTimer* + si-NC. Scaler bar, 10 μm. Green represents newly synthesized mitochondria, red represents mature mitochondria. Images were analyzed by Leica LAS X life science software. **g** The expression of genes involved in PGC1α-NRF1-TFAM signaling pathway was measured by RT-qPCR at 48 h after transfection with si-*GHR* and si-NC. **h** The expression of mtDNA encoded genes was measured by RT-qPCR at 48 h after transfection with si-*GHR* and si-NC. **i** The relative mtDNA content was measured by RT-qPCR at 48 h after transfection with si-*GHR* and si-NC. **j-l** Western blots with anti-GHR, anti-PGC1α, anti-NRF1 and anti-β-actin at 48 h after transfection with si-*GHR* and si-NC. Data are shown as mean ± SEM, *p < 0.05, **p < 0.01

We asked whether local *GHR* regulates mitochondrial biogenesis during myoblast differentiation using *GHR* knockdown and overexpression. Knockdown or overexpression efficiency was measured by RT-qPCR and western blot (Fig. 1a, j, k; S2a, j, k). Regarding GH-GHR-IGFs axis, *GHR* knockdown decreased the expression of *IGF1*, but did not alter the expression of *GH* and *IGF2* (Fig. 1b), indicating that *GHR* might exert its downstream roles through auto/paracrine *IGF1*. On the contrary, *GHR* overexpression promoted the expression of *GH* and *IGF1* (Fig. S2b), indicating a positive feedback regulation mechanism.

Next, we investigated the effects of *GHR* on mitochondrial biogenesis by first measuring mitochondrial mass using MTG staining. *GHR* knockdown reduced mitochondrial mass (Fig. 1c, d), while overexpression of *GHR* was accompanied by an increase in mitochondrial mass (Fig. S2c, d). To further verify the effects of *GHR* on mitochondrial biogenesis during myoblast differentiation, we utilized a *MitoTimer* reporter gene. p*MitoTimer* expresses a mitochondrial targeted DsRed protein that changes from green fluorescence to red in mitochondria within 48 h; therefore, it can help to distinguish newly synthesized mitochondria from mature mitochondria [35]. *GHR* knockdown increased the ratio of red signal to green signal (Fig. 1e, f), while overexpression of *GHR* decreased this ratio (Fig. S2e, f), indicating that *GHR* promotes the synthesis of new mitochondria. We then examined the expression of *PGC1α*, *NRF1* and *TFAM*, which are markers of mitochondrial biogenesis, after transfection. *GHR* knockdown decreased the expression of *PGC1α*, *NRF1* and *TFAM* (Fig. 1g), while *GHR* overexpression had the opposite effects (Fig. S2g). We also investigated the effects of *GHR* on mtDNA transcription and replication. *GHR* knockdown repressed the expression of mtDNA encoded gene (represented by *ND1*、*CYTB*、*COX1*、*ATP6*), and mtDNA copy number (represented by *ND1* and *tRNA-Leu*) (Fig. 1h, i). Opposite results were found after we overexpressed *GHR* (Fig. S2h, i). Finally, we examined the protein levels of mitochondrial biogenesis markers. *GHR* knockdown decreased the protein level of PGC1α, NRF1 and TOMM20 (Fig. 1j, l), while overexpression of *GHR* increased the protein level of these markers (Fig. S2j, l). Taken together, these results suggest that local *GHR* promotes mitochondrial biogenesis during myoblast differentiation.

### *IGF1* promotes mitochondrial biogenesis

**Fig. 2:**
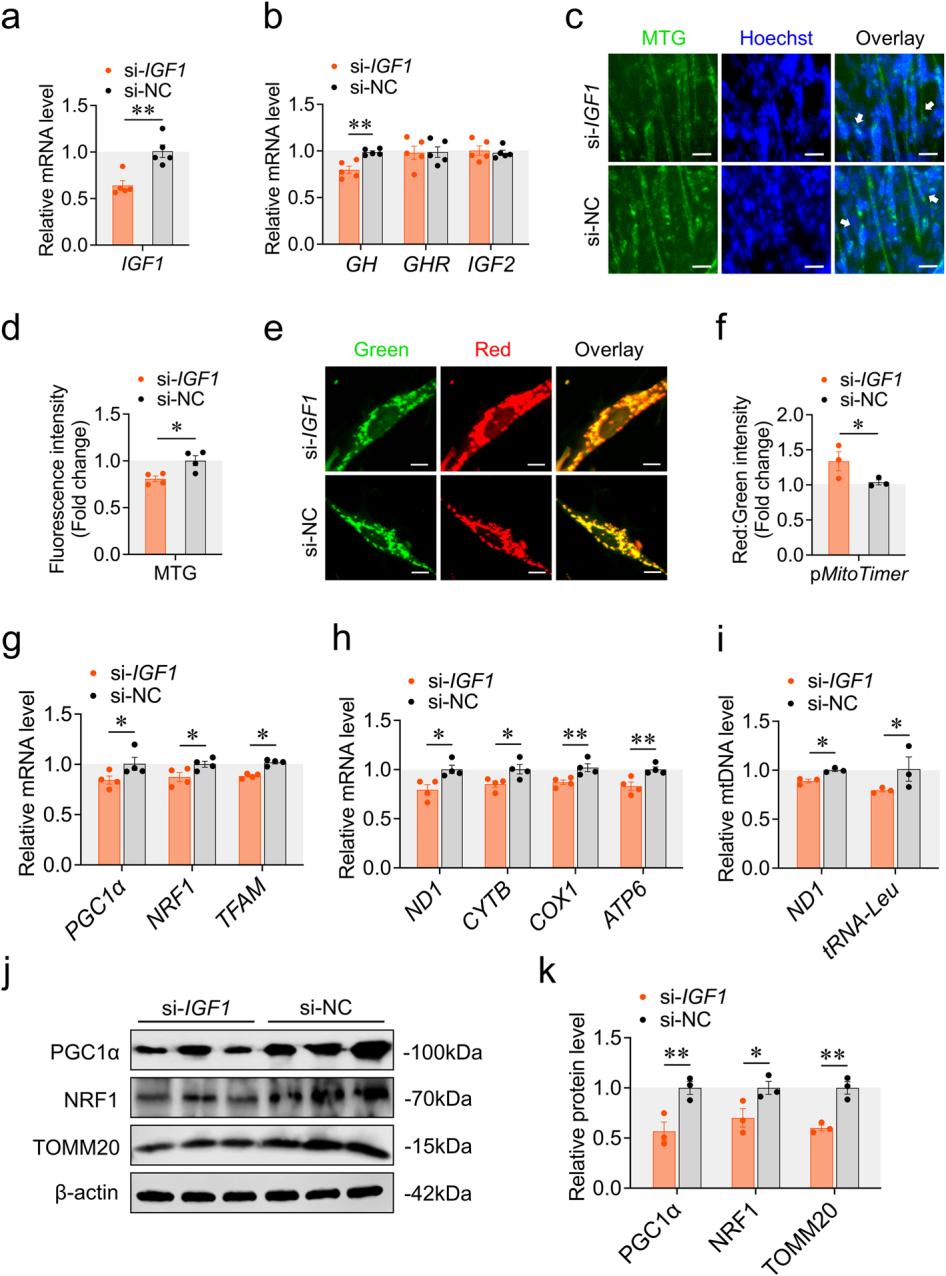
*IGF1* knockdown inhibits mitochondrial biogenesis. **a** Knockdown efficiency was measured by RT-qPCR at 48 h after transfection with si-*IGF1* and si-NC. **b** The expression of genes involved in the GH-GHR-IGFs signaling pathway was measured by RT-qPCR at 48 h after transfection with si-*IGF1* and si-NC. **c** and **d** MTG staining of CPM was measured at 48 h after transfection with si-*IGF1* and si-NC. White arrow labeled elongated myoblasts. Scaler bar, 25 μm. **e** and **f** Confocal images were observed at 48 h after co-transfection with p*MitoTimer* + si-*IGF1* and p*MitoTimer* + si-NC. Scaler bar, 10 μm. Green represents newly synthesized mitochondria, red represents mature mitochondria. Images were analyzed by Leica LAS X life science software. **g** The expression of genes involved in PGC1α-NRF1-TFAM signaling pathway was measured by RT-qPCR at 48 h after transfection with si-*IGF1* and si-NC. **h** The expression of mtDNA encoded genes was measured by RT-qPCR at 48 h after transfection with si-*IGF1* and si-NC. **i** The relative mtDNA content was measured by RT-qPCR at 48 h after transfection with si-*IGF1* and si-NC. **j** and **k** Western blots with anti-PGC1α, anti-NRF1 and anti-β-actin at 48 h after transfection with si-*IGF1* and si-NC. Data are shown as mean ± SEM, *p < 0.05, **p < 0.01

We then asked whether auto/paracrine produced IGF1 affects mitochondrial biogenesis during myoblast differentiation using *IGF1* knockdown and overexpression. Knockdown or overexpression efficiency was measured by RT-qPCR (Fig. 2a; S3a). Regarding GH-GHR-IGFs axis, *IGF1* knockdown decreased the expression of *GH*, but did not alter the expression of *GHR* and *IGF2* (Fig. 2b), indicating a positive feedback regulation mechanism involved in *IGF1* roles. Notably, *IGF1* overexpression repressed the expression of *GH, GHR* and *IGF2* (Fig. S3b), indicating a negative feedback regulation mechanism that is opposite to the results of *IGF1* knockdown.

Like the results acquired with *GHR* above, *IGF1* knockdown reduced mitochondrial mass (Fig. 2c, d), while overexpression of *IGF1* was accompanied by an increase in mitochondrial mass (Fig. S3c, d). As for MitoTimer reporter system, *IGF1* knockdown increased the ratio of red signal to green signal (Fig. 1e, f), while overexpression of *IGF1* decreased this ratio (Fig. S2e, f), indicating that *IGF1* promotes the synthesis of new mitochondria. Accordingly, *IGF1* knockdown decreased the expression of *PGC1α*, *NRF1* and *TFAM* (Fig. 2g), while *IGF1* overexpression had the opposite effects (Fig. S2g). In parallel, *IGF1* knockdown repressed the expression of mtDNA encoded gene (represented by *ND1*、*CYTB*、*COX1*、*ATP6*), and mtDNA copy number (represented by *ND1* and *tRNA-Leu*) (Fig. 2h, i). Opposite results were found after we overexpressed *IGF1* (Fig. S3h, i). Additionally, we examined the protein level of mitochondrial biogenesis markers. *IGF1* knockdown decreased the protein level of PGC1α, NRF1 and TOMM20 (Fig. 2j, k), while overexpression of *IGF1* increased the protein level of these markers (Fig. S3j, k). Altogether, these results suggest that *IGF1* promotes mitochondrial biogenesis during myoblast differentiation.

### Regulation of mitochondrial biogenesis by *GHR* depends on *IGF1*

**Fig. 3:**
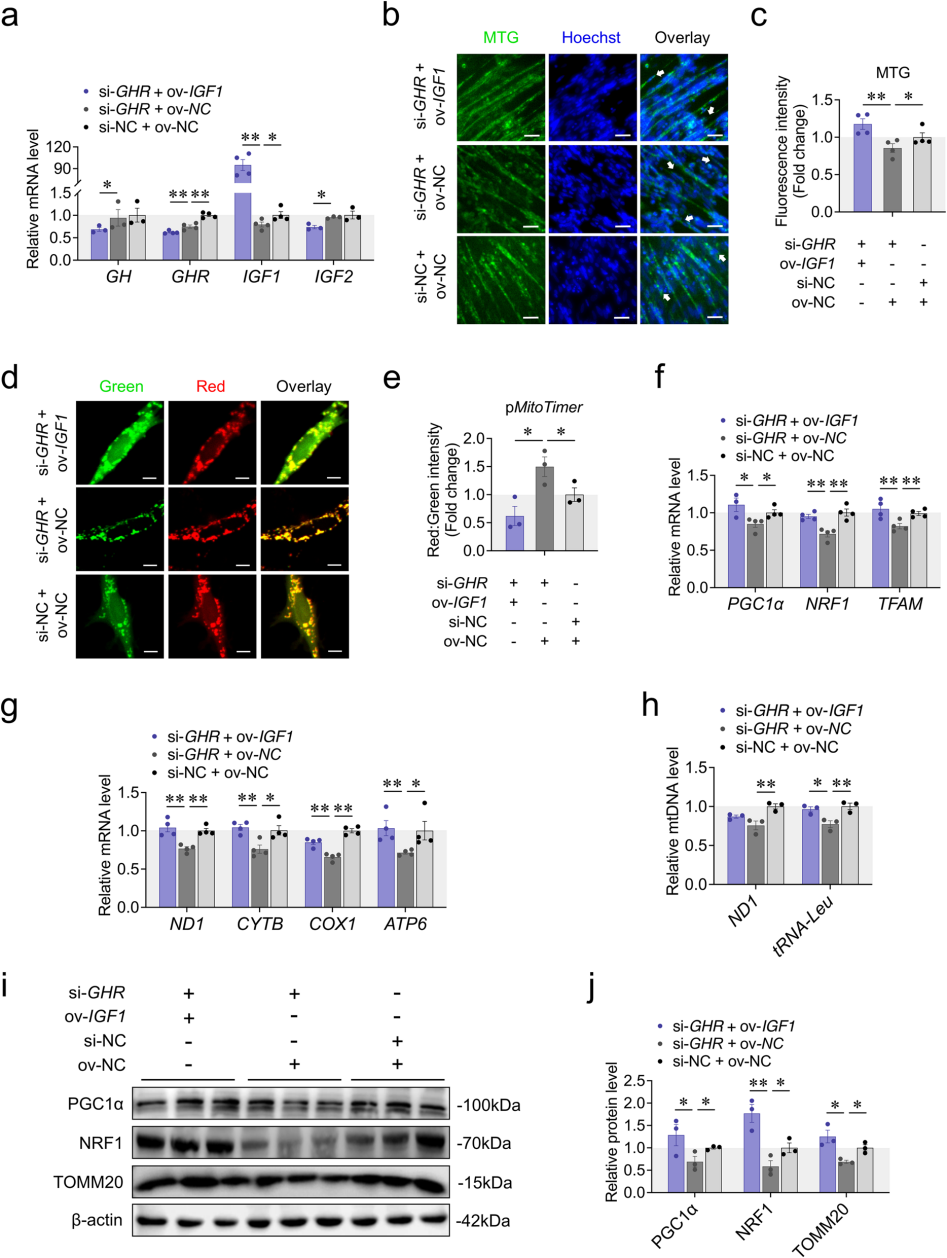
Regulation of mitochondrial biogenesis by *GHR* depends on *IGF1*. **a** The expression of genes involved in the GH-GHR-IGFs signaling pathway was measured by RT-qPCR at 48 h after co-transfection with si-*GHR* + pcDNA3.1-*IGF1*, si-*GHR* + pcDNA3.1 and si-NC + pcDNA3.1. **b** and **c** MTG staining of CPM was measured at 48 h after transfection with co-transfection with si-*GHR* + pcDNA3.1-*IGF1*, si-*GHR* + pcDNA3.1 and si-NC + pcDNA3.1. White arrow labeled elongated myoblasts. Scaler bar, 25 μm. **d** and **e** Confocal images were observed at 48 h after co-transfection with p*MitoTimer* + si-*GHR* + pcDNA3.1-*IGF1*, + si-*GHR* + pcDNA3.1 and + si-NC + pcDNA3.1. Scaler bar, 10 μm. Green represents newly synthesized mitochondria, red represents mature mitochondria. Images were analyzed by Leica LAS X life science software. **f** The expression of genes involved in PGC1α-NRF1-TFAM signaling pathway was measured by RT-qPCR at 48 h after co-transfection with si-*GHR* + pcDNA3.1-*IGF1*, si-*GHR* + pcDNA3.1 and si-NC + pcDNA3.1. **g** The expression of mtDNA encoded genes was measured by RT-qPCR at 48 h after co-transfection with si-*GHR* + pcDNA3.1-*IGF1*, si-*GHR* + pcDNA3.1 and si-NC + pcDNA3.1. **h** The relative mtDNA content was measured by RT-qPCR at 48 h after co-transfection with si-*GHR* + pcDNA3.1-*IGF1*, si-*GHR* + pcDNA3.1 and si-NC + pcDNA3.1. **i** and **j** Western blots with anti-PGC1α, anti-NRF1 and anti-β-actin at 48 h after co-transfection with si-*GHR* + pcDNA3.1-*IGF1*, si-*GHR* + pcDNA3.1 and si-NC + pcDNA3.1. Data are shown as mean ± SEM, *p < 0.05, **p < 0.01

The tight connection between *GHR* and *IGF1* along with the synchronous results prompted us asked whether *IGF1* could mitigate the impairments of *GHR* knockdown on mitochondrial biogenesis. Therefore, we knocked down *GHR* in presence and absence of *IGF1* overexpression during myoblast differentiation. The expression of genes involved in GH-GHR-IGFs axis were coincided with the results as above (Fig. 3a). Using MTG staining, MitoTimer reporter system, RT-qPCR and western blots, we found that overexpression of *IGF1* completely restored or reversed the impairments of *GHR* knockdown on mitochondrial biogenesis (Fig. 3b-j), indicating that the regulation of mitochondrial biogenesis by *GHR* depends on *IGF1*.

### Local *GHR* regulates mitochondrial biogenesis via IGF1-PI3K/AKT/CREB pathway

**Fig. 4:**
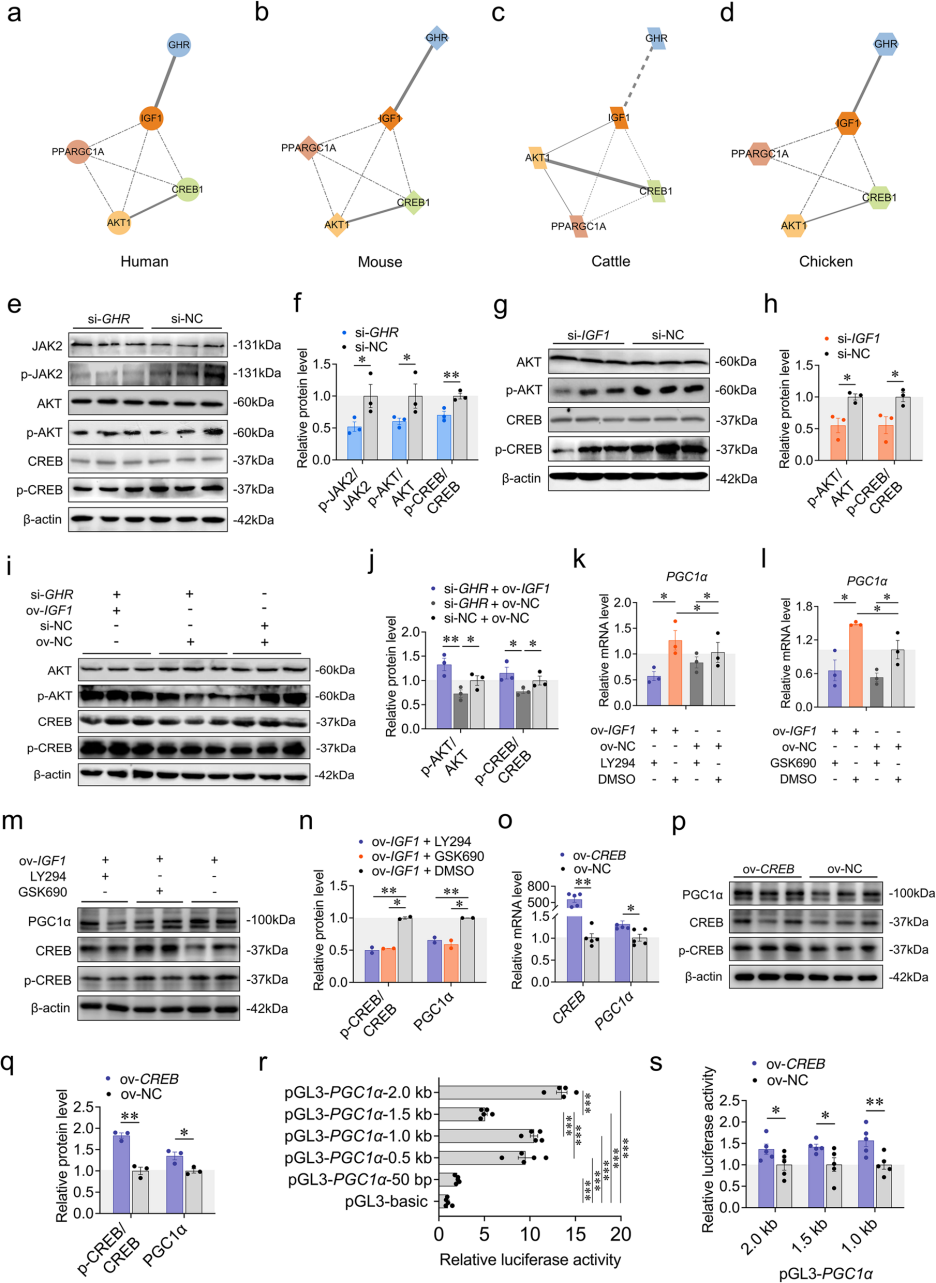
Local *GHR* regulates mitochondrial biogenesis via IGF1-PI3K/AKT/CREB pathway. **a** The PPI network of human GHR, IGF1, AKT, CREB and PGC1α. **b** The PPI network of mouse GHR, IGF1, AKT, CREB and PGC1α. **c** The PPI network of pig GHR, IGF1, AKT, CREB and PGC1α. **d** The PPI network of chicken GHR, IGF1, AKT, CREB and PGC1α. Protein–protein interaction was performed by the String database and visualized by Cytoscape (version 3.4.0). **e** and **f** Western blots with anti-JAK2, anti-p-JAK2, anti-AKT1, anti-p-AKT1, anti-CREB1, anti-p-CREB1 and anti-β-actin at 48 h after transfection with si-*GHR* and si-NC. **g** and **h** Western blots with anti-AKT1, anti-p-AKT1, anti-CREB1, anti-p-CREB1 and anti-β-actin at 48 h after transfection with si-*IGF1* and si-NC. **i** and **j** Western blots with anti-AKT1, anti-p-AKT1, anti-CREB1, anti-p-CREB1 and anti-β-actin at 48 h after co-transfection with si-*GHR* + pcDNA3.1-*IGF1*, si-*GHR* + pcDNA3.1 and si-NC + pcDNA3.1. **k** LY294002 (PI3K inhibitor) was added at 24 h before measuring the expression of *PGC1α* by RT-qPCR at 48 h after transfection of pcDNA3.1-*IGF1* and pcDNA3.1. **l** GSK690693 (AKT inhibitor) was added at 24 h before measuring the expression of *PGC1α* by RT-qPCR at 48 h after transfection of pcDNA3.1-*IGF1* and pcDNA3.1. **m** and **n** LY294002 or GSK690693 was added at 24 h before measuring the protein levels with anti-PGC1α, anti-CREB1, anti-p-CREB1 and anti-β-actin at 48 h after transfection of pcDNA3.1-*IGF1* and pcDNA3.1. **o** The expression of *CREB* and *PGC1α* was measured by RT-qPCR at 48 h after transfection with pcDNA3.1-*CREB* and pcDNA3.1. **p** and **q** Western blots with anti-PGC1α, anti-CREB1, anti-p-CREB1 and anti-β-actin at 48 h after transfection with pcDNA3.1-*CREB* and pcDNA3.1. **r** Dual-Luciferase report assays transfected with reporter vectors containing different length of 5′ upstream region of *PGC1α*. **s** Dual-Luciferase report assays of *CREB* overexpression co-transfected with reporter vectors containing different length of 5′ upstream region of *PGC1α.* Data are shown as mean ± SEM, *p < 0.05, **p < 0.01

Next, we aimed to identify the underlying mechanism by which local *GHR* promotes mitochondrial biogenesis during myoblast differentiation. GH combines with GHR to regulate IGF1 production via the JAK2/STAT5 pathway through endocrine and paracrine/autocrine mechanisms [36, 37]. IGF1 binds to its receptor to activate the PI3K/AKT signaling pathway in murine fibroblasts [38]. AKT regulates the phosphorylation of the transcription factor CREB to promote human 293 T cells survival [39]. Phosphorylated CREB further activates downstream *PGC1α* transcription through cAMP response element in human hepatoma HepG2 cells [40]. By using the String database, there is indeed a potential protein–protein interaction (PPI) network among GHR, IGF1, AKT, CREB and PGC1α in diverse species (Fig. 4a-d). Based on these foregoing results, we wondered if local *GHR* might regulate mitochondrial biogenesis via IGF1-PI3K/AKT/CREB pathway during myoblast differentiation.

To test this hypothesis, we first determined the expression levels of related proteins involved in this pathway by western blots. *GHR* knockdown decreased the protein level of p-JAK2、p-AKT and p-CREB (Fig. 4e, f), while *GHR* overexpression increased the protein levels (Fig. S4a, b). Likewise, *IGF1* knockdown decreased the protein level of p-AKT and p-CREB (Fig. 4g, h), while *IGF1* overexpression had an opposite result (Fig. S4c, d). Moreover, overexpression of *IGF1* also reversed the effects of reduced protein level in *GHR* knockdown cells (Fig. 4i, j).

To further evaluate if the effect of *IGF1* on mitochondrial biogenesis was transmitted through PI3K/AKT pathway, we treated the cells with LY294002 (PI3K inhibitor) or GSK690693 (AKT inhibitor) after *IGF1* overexpression during myoblast differentiation. We observed that these PI3K/AKT pathway inhibitors significantly decreased CREB protein phosphorylation level (Fig. 4m, n). Among the same line, *PGC1α* mRNA and protein expression were both decreased after blunting the PI3K/AKT pathway (Fig. 4k-n).

We next manipulated *CREB* expression to examine whether *PGC1α* expression was regulated by this transcription factor during myoblast differentiation. The results revealed that, as expected, significant increase of *PGC1α* expression and its protein level after *CREB* overexpression (Fig. 4o-q). Further investigation using dual-luciferase assays transfected with reporter vectors containing different lengths of 2000 bp upstream region of *PGC1α* (Fig. 4r). Dual-luciferase assays revealed that significantly increased luciferase activity in different pGL3-PGC1α reporter vectors in *CREB* overexpressed cells, indicating that CREB directly promoted the transcription of *PGC1α* by acting on the proximal promoter region (Fig. 4s). Inversely, *CREB* knockdown had the opposite effects (Figure S4e-h). Taken together, we demonstrate that local *GHR* regulates mitochondrial biogenesis via IGF1-PI3K/AKT/CREB pathway during myoblast differentiation.

### Local *GHR *enhances mitochondrial function through *IGF1*

**Fig. 5:**
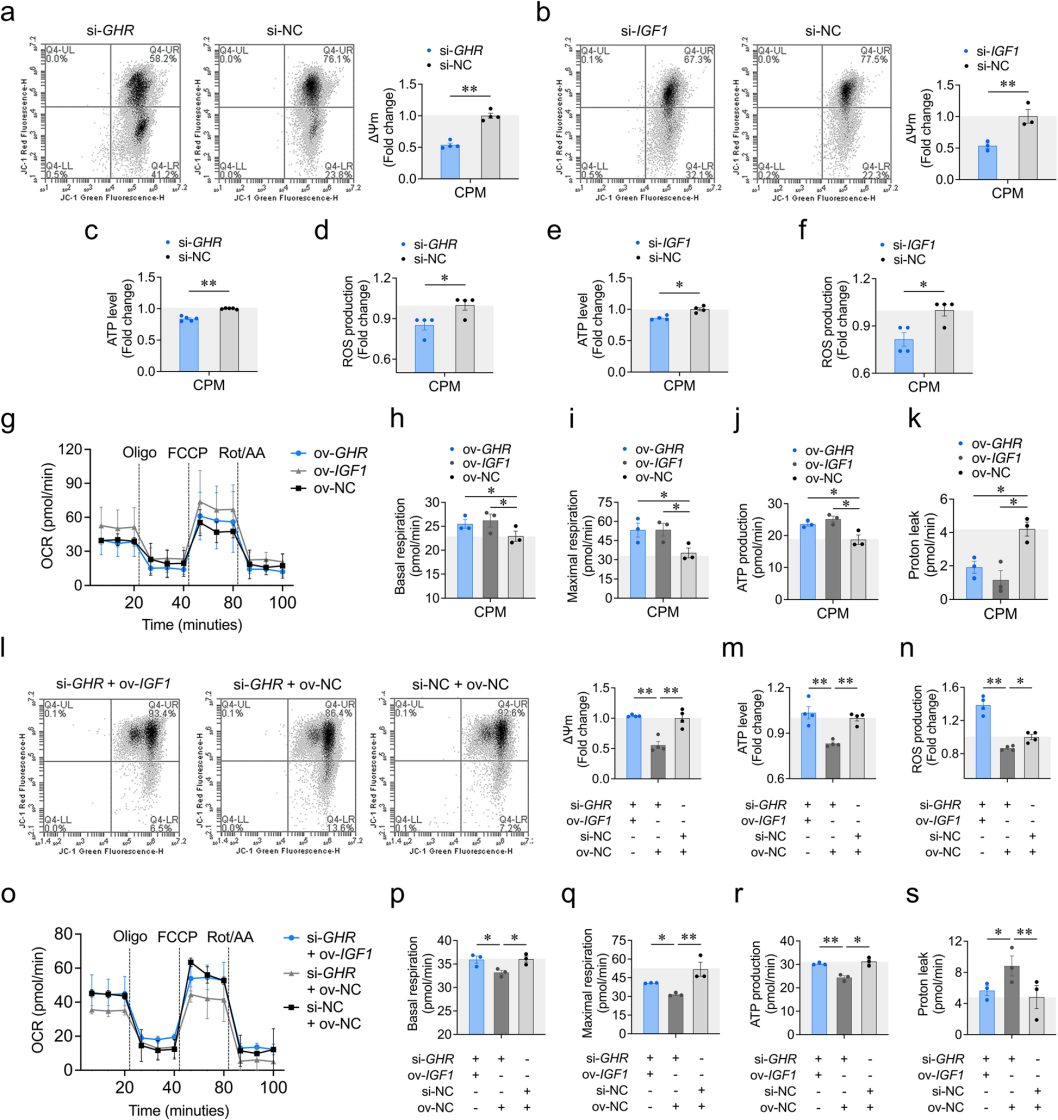
Local *GHR* enhances mitochondrial function through *IGF1*. **a** ΔΨm were measured by the fluorescence of JC-1 at 48 h after transfection with si-*GHR* and si-NC. **b** ΔΨm were measured by the fluorescence of JC-1 at 48 h after transfection with si-*IGF1* and si-NC. **c** ATP level was measured at 48 h after transfection with si-*GHR* and si-NC. **d** Reactive oxygen species production was measured by the fluorescence of DCF at 48 h after transfection with si-*GHR* and si-NC. **e** ATP level was measured at 48 h after transfection with si-*IGF1* and si-NC. **f** Reactive oxygen species production was measured by the fluorescence of DCF at 48 h after transfection with si-*IGF1* and si-NC (shared control group with si-*GHR*). **g** Oxygen consumption rate (OCR) was measured in CPM utilizing Seahorse Extracellular Flux Analyzer after transfection with pcDNA3.1-*GHR*, pcDNA3.1-*IGF1* and pcDNA3.1. Dotted lines indicate when mitochondrial inhibitors were added. **h** Basal respiration and **i** Maximal respiration were determined following mitochondrial uncoupling by FCCP. **j** ATP-production was assessed after inhibition of ATP synthase by oligomycin. **k** Proton leakage was determined after inhibiting complex I and III by rotenone (rot) and antimycin-A (AA). **l** ΔΨm was measured by the fluorescence of JC-1 at 48 h after co-transfection with si-*GHR* + pcDNA3.1-*IGF1*, si-*GHR* + pcDNA3.1 and si-NC + pcDNA3.1. **m** ATP level was measured at 48 h after co-transfection with si-*GHR* + pcDNA3.1-*IGF1*, si-*GHR* + pcDNA3.1 and si-NC + pcDNA3.1. **n** Reactive oxygen species production was measured by the fluorescence of DCF at 48 h co-transfection with si-*GHR* + pcDNA3.1-*IGF1*, si-*GHR* + pcDNA3.1 and si-NC + pcDNA3.1. **o** OCR was measured in CPM utilizing Seahorse Extracellular Flux Analyzer after co-transfection with si-*GHR* + pcDNA3.1-*IGF1*, si-*GHR* + pcDNA3.1 and si-NC + pcDNA3.1. **p-s** Measured and calculated parameters of mitochondrial respiration. Data are shown as mean ± SEM, *p < 0.05, **p < 0.01

Mitochondrial biogenesis plays an essential role in maintaining normal mitochondrial OXPHOS, raising the question of whether inhibited mitochondrial biogenesis by *GHR* knockdown could ultimately result in impaired mitochondrial function during myoblast differentiation. To explore this, we first performed mitochondrial membrane potential, luminescence-based ATP level and ROS production assay to evaluate mitochondrial function. *GHR* knockdown impaired mitochondrial function, as indicated by reduced ΔΨm and ATP level as well as ROS production (Fig. 5a, c, d). However, *GHR* overexpression enhanced mitochondrial function (Fig. S5a, c, d). Same consequences were observed after *IGF1* knockdown or overexpression (Fig. 5b, e, f; S5b, e, f). Furthermore, *IGF1* overexpression ameliorated the effects of *GHR* knockdown on mitochondrial function (Fig. 5l-m).

On the other hand, we measured mitochondrial OXPHOS utilizing Seahorse Extracellular Flux Analyzer after *GHR* and *IGF1* overexpression to recapitulate the results above. We found that *GHR* and *IGF1* overexpression enhanced oxygen consumption rate (OCR), with the control groups showing lower basal and maximal respiration (Fig. 5g-i). *GHR* and *IGF1* overexpression also had higher ATP production as measured by inhibition of ATP synthase with oligomycin, which led to smaller decrease in the basal respiration of control groups (Fig. 5j). Meanwhile, *GHR* and *IGF1* overexpression repressed proton leakage as measured by inhibiting complex I and III by rotenone and antimycin-A, which may explain the higher ROS production with enhanced mitochondrial function under normal physiology condition (Fig. 5k). Consistently, *IGF1* overexpression partially alleviated the effects of *GHR* knockdown on mitochondrial respiration (Fig. 5o-s). Altogether, these results suggest that local *GHR* enhances mitochondrial function through *IGF1* during myoblast differentiation.

### *GHR* knockdown represses myoblast differentiation

**Fig. 6:**
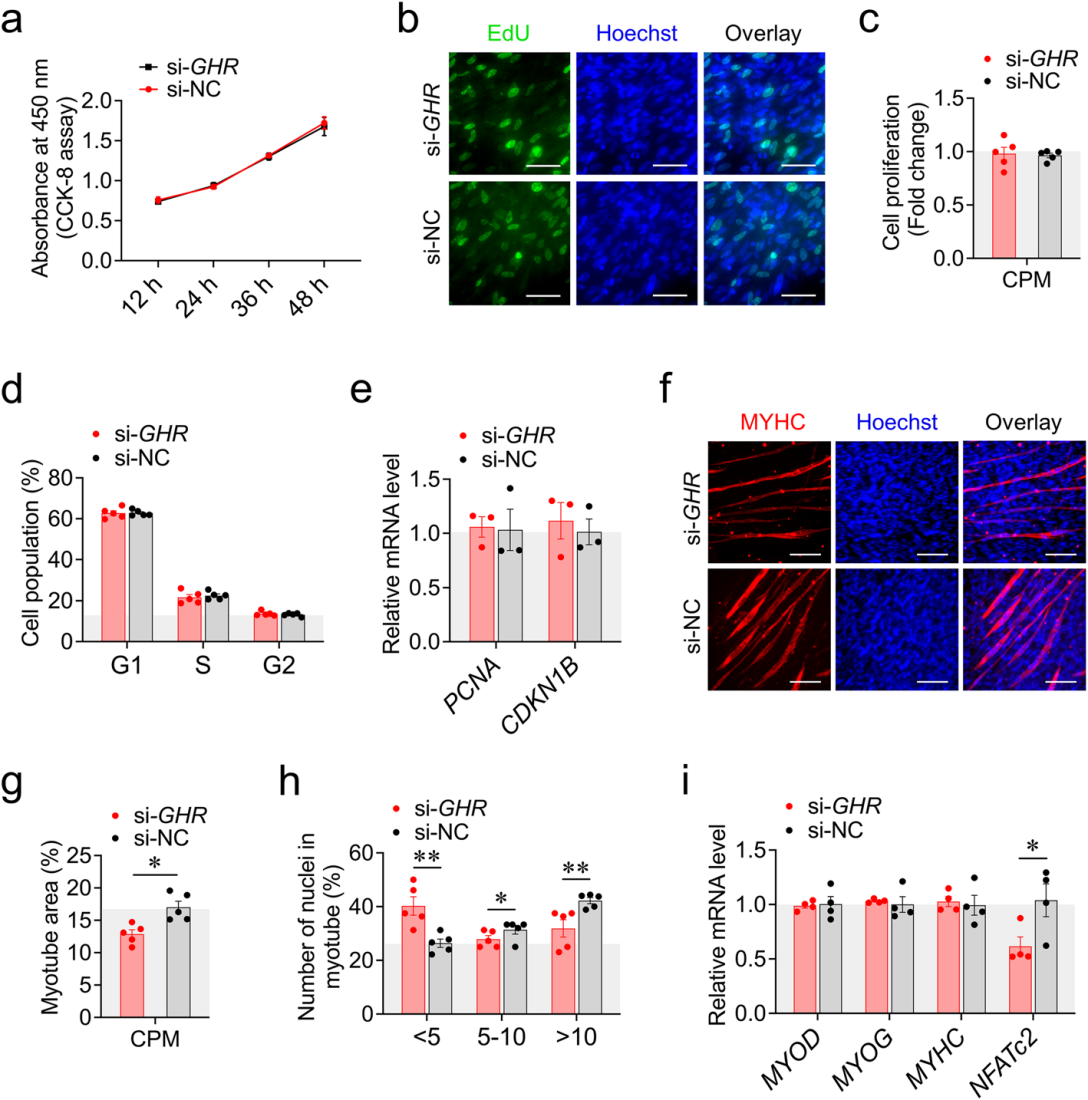
*GHR* knockdown represses myoblast differentiation. **a** CCK-8 assays were performed after transfection with si-*GHR* and si-NC. **b** and **c** EdU proliferation assays were performed after transfection with si-*GHR* and si-NC. **d** Cell cycle analysis were performed after transfection with si-*GHR* and si-NC. **e** The expression of cell proliferation marker genes was measured by RT-qPCR at 48 h after transfection with si-*GHR* and si-NC. **f–h** MyHC staining, myotube area and myoblast fusion index were measured at 48 h after transfection with si-*GHR* and si-NC. **i** The expression of myoblast differentiation marker genes was measured by RT-qPCR at 48 h after transfection with si-*GHR* and si-NC. Data are shown as mean ± SEM, *p < 0.05, **p < 0.01

Next, we sought to understand the effects of local *GHR* on myoblast proliferation and differentiation. Using CCK-8 and EdU assays, we found that *GHR* knockdown or overexpression did not alter myoblast proliferation cultured in GM (Fig. 6a-c; S6a-c). This is further supported by the fact that *GHR* had no influence on the number of cells in G0, S and G1 phase and the expression of cell proliferation marker genes (Fig. 6d, e; S6d, e), indicating that *GHR* does not affect myoblast proliferation.

Previous studies have found that GH signaling affects myoblast development by stimulating accumulation of additional myonuclei into nascent myotubes (myoblast fusion) induced by NFATc2 in a cell-autonomous manner [14, 15]. Similarly, our *GHR* knockdown repressed the formation of myotubes, decreased the proportion of myotubes with more than ten nuclei, and inhibited the expression of *NFATc2* in CPM (Fig. 6f-i). Of note, we unexpectedly found that *GHR* overexpression also repressed myoblast differentiation (Fig. S6f-i). These results suggest that *GHR* knockdown represses myoblast differentiation.

## Discussion

**Fig. 7:**
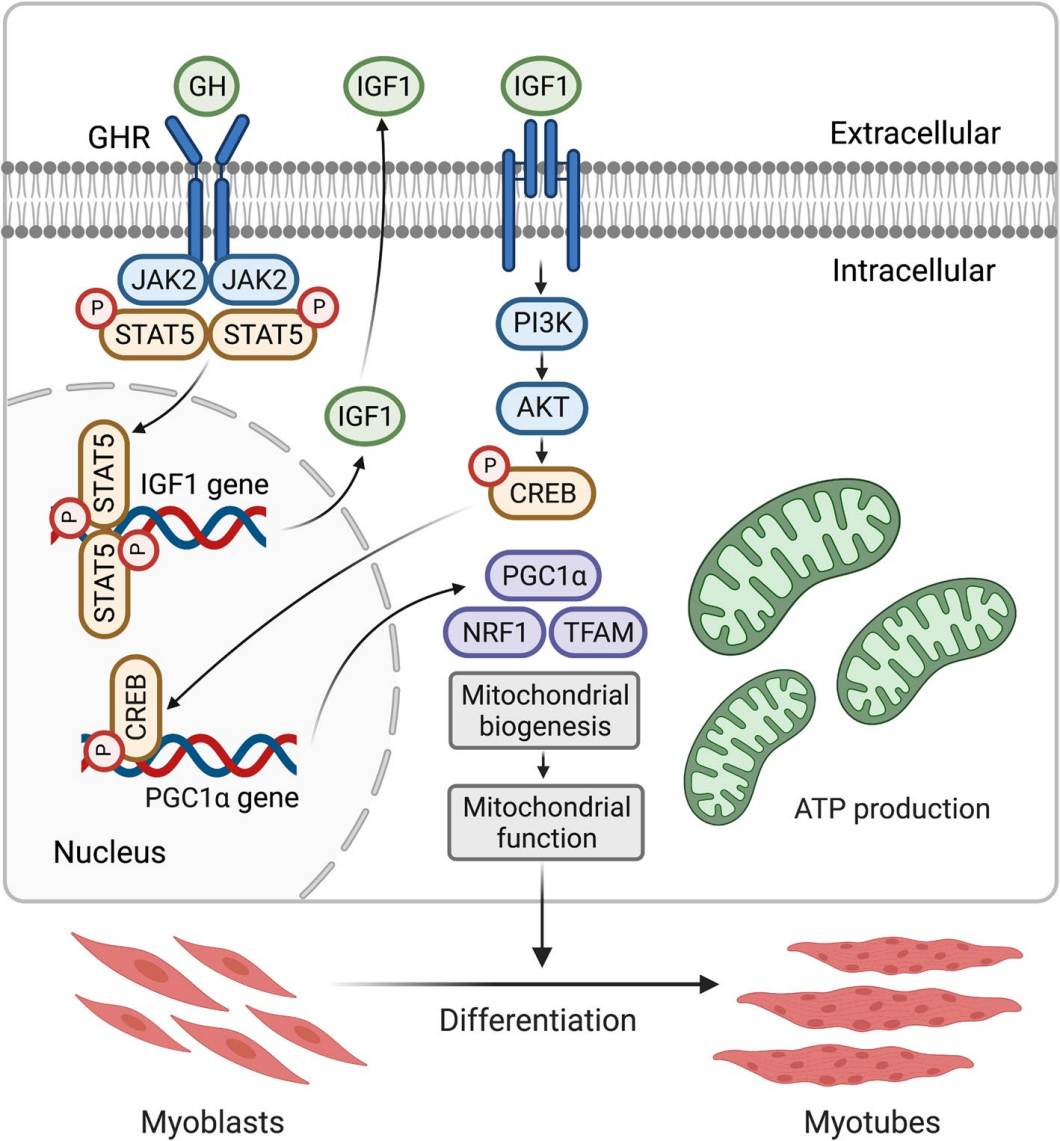
Schematic diagram for the mechanistic model of the *GHR* roles in regulation of mitochondrial function during myoblast differentiation. Local *GHR* enhances mitochondrial function by promoting mitochondrial biogenesis via *IGF1*-PI3K/AKT/CREB pathway during myoblast differentiation. This graphical abstract was created with Biorender.com

At present, canonical GH-GHR-IGF1 axis has been advanced for more than a century, and mostly focuses on the treatment of growth-related disorders [7]. However, the relationship between local GH-GHR-IGF1 axis and mitochondria during the muscle development is rarely reported. Our previous research revealed that mutations in *GHR* elicit a decrease in the number and diameter of muscle fibers in E14 and 7w SLD chicken [16], indicating that *GHR* may affect the growth and development of skeletal muscle in the embryonic stage. Myoblasts play a central role in the formation and growth of skeletal muscle. Accordingly, we sought to unveil the molecular mechanism of local *GHR* regulating mitochondrial biogenesis during myoblast differentiation, and obtained evidence that local *GHR* regulates muscle development in the embryonic stage from the perspective of mitochondria (Fig. 7). It may provide a theoretical basis for the development of inhibitor or activator molecules targeting mitochondria to promote muscle development.

It is well known that myogenesis is a sophisticated process. In mammals, myoblasts first differentiate to primary fibers during embryonic stage (E10.5-E12.5); then, myoblasts and single myoblasts fuse to the existing primary fibers to generate secondary myofibers during fetal stage (E14.5-P0); at last, satellite cell proliferation and fusion with existing myofibers result in myofiber growth by rapid increase in myonuclear number during neonatal stage (P0-P21). Consistently, chicken skeletal muscle mainly remains in the period of proliferation and differentiation before E16, and the fusion of muscle fibers is basically completed after E18. *GHR* is highly expressed during E10-E14, but is relatively low after E18 in leg muscle [34]. In present study, *GHR* expression was relatively high in the differentiation phase and gradually up-regulated during myoblast differentiation. These results suggest that *GHR* mainly plays its roles during myoblast differentiation. Knockout of *GHR* or addition of GH does not affect the proliferation of myoblasts after 8 h [14, 15]. Here, *GHR* knockdown or overexpression had no impact on myoblast proliferation, further indicating that *GHR* does not account for the process of myoblast proliferation. On the other hand, mitochondrial OXPHOS remains continuously inhibited during myoblast proliferation, but is highly fired during myoblast differentiation. Based on the above research results, we thereby explored the molecular mechanism of *GHR* regulating mitochondrial function during myoblast differentiation phase.

*IGF1* sequence is highly conserved among species, whereas *IGF2* is different [41]. Like the *GHR* expression profile, *IGF1* is highly induced during E15-E18 in chicken leg muscles, but is relatively low after E18; while *IGF2* is relatively high expression after E18 [42]. *IGF1* was significantly up-regulated after *GHR* overexpression and down-regulated after *GHR* knockdown during myoblast differentiation, while *IGF2* expression did not change. These results indicate that *GHR* mainly played its regulatory roles through endogenous *IGF1* rather than *IGF2* in the embryonic stage. It is generally believed that IGFs are mainly induced by *GHR* in the liver to control the growth and development of animals. However, due to the embryo still lacking a mature circulatory system, IGFs must play their regulatory role through paracrine or autocrine mechanisms in the early embryonic development (before E15-E16) [43]. IGF1 produced by the liver is not necessary for body growth after birth, GH mainly promotes body growth after birth through autocrine IGF1 in non-liver tissues [44]. Although *GHR* is scarcely expressed in myoblasts, local *GHR* is likely to act as a control valve during myogenesis. Compliance with the results from Segard et al. (2003), *GHR* overexpression increased the expression of *GH* and *IGF1*, suggesting that GH and IGF1 regulate embryonic myoblast development through an auto/paracrine manner. Besides, *IGF1* knockdown had no effect on *IGF2* expression, while *IGF1* overexpression down-regulated *IGF2* expression, implying that there may be a competing relationship between *IGF1* and *IGF2*.

Mitochondrial biogenesis supports the normal number, structure, and function of mitochondria, which is mainly regulated by nuclear genes through the PGC1α-NRF1-TFAM signaling pathway [45–49]. PGC1α is a member of the PGC1 family, which also includes PGC1β and PRC. PGC1α acts as a mediator of mitochondrial biogenesis under different physiological conditions, whereas the role of PGC1β is limited to the maintenance of basal mitochondrial function. By contrast, PRC function appears to be restricted to the regulation of gene expression in proliferating cells [48]. These co-activators coordinate with nuclear respiration factors NRF1 and NRF2 to regulate the *TFAM* expression thereby affecting mtDNA replication and transcription [50]. Notably, *TFAM* expression does not always exhibit parallel with the mtDNA copy number, TFAM should be used judiciously as a marker of mitochondrial biogenesis [51]. There is evidence that the transcription level of *TFAM* increased along with the up-regulation of mtDNA during the differentiation process of myoblasts [52], suggesting that *TFAM* is a suitable marker for mitochondrial biogenesis during myoblast differentiation. Therefore, the expression of *PGC1α*, *NRF1*, *TFAM* and mitochondrial-related gene (*ND1*, *CYTB*, *COX1*, *ATP6*) as well as mtDNA copy number were selected as the main indicators for measuring mitochondrial biogenesis. *NRF1* and *TFAM* expression are reduced in the skeletal muscle of GHRKO mice [53]. The expression of mitochondrial biogenesis markers (*PGC1α*, *NRF1*, *TFAM*) and mtDNA encoded OXPHOS gene are all down-regulated in SLD chicken skeletal muscle [54]. These results are consistent with our study, indicating that *GHR* plays a positive role in regulating mitochondrial biogenesis. We also revealed that *IGF1* promoted mitochondrial biogenesis during myoblast differentiation, coinciding with the prior results that have been well summarized in our previous review [32]. Additionally, improving mitochondrial biogenesis is generally regarded as an ideal method to enhance cell function, which even can be used as a potential mitochondrial therapy [55]. Thus, we suggest that the GH-GHR-IGF1 axis may be used as a potential molecular target to promote mitochondrial biogenesis in the future.

The great complexity of the *GHR* on mitochondrial function is mirrored by the divergent results from a wide range of models*. GHR* knockout is harmful to the mitochondrial function of bone cells and fibroblasts [56]. Mitochondrial function is impaired in SLD chicken skeletal muscle [54]. We also revealed that *GHR* enhanced mitochondrial function during myoblast differentiation. On the contrary, mitochondrial function of elderly GHRKO mice is enhanced in the liver, muscle, heart, kidney, and brain; three TCA cycle enzymes abundance (isocitrate dehydrogenase, fumarase and malate dehydrogenase) in the proteome of GHRKO pig liver is significantly increased, indicating that *GHR* inhibits mitochondrial function in vivo [57, 58]. On the other hand, the effects of *IGF1* on mitochondrial biogenesis and mitochondrial function exhibited the consistent results, indicating that *IGF1* enhances mitochondrial function by regulating mitochondrial biogenesis in vitro. Counterintuitively, the ROS production did not increase with the reduction in ΔΨm, but showed consistency with the trend of ΔΨm in our present results. This may be explained by that mitochondrial proton leakage (due to the reduced ΔΨm) can offset the ROS production under various physiological and pathological conditions to protect cells from oxidative stress, resulting in a positive correlation between ΔΨm and ROS production [59, 60]. Similarly, there is evidence that muscle mitochondrial dysfunction can be fired by a range of factors, but not all of them are decided by ROS production [61].

Mitochondria provide ATP for the differentiation process of myoblasts, and mitochondrial dysfunction impairs the myoblast differentiation. Accordingly, mitochondrial biogenesis controls the energy supply requirements of myotubes [62]. In C2C12, reduced *PGC1α* expression increases ROS production, mitochondrial damage, mitophagy, and ultimately inhibits the myoblast differentiation [26]. Consistent with the results of Sotiropoulos et al. (2006), *GHR* knockdown repressed the differentiation process of myoblast. According to the results above, we perceive that *GHR* knockdown inhibits mitochondrial biogenesis and further impairs mitochondrial function, resulting in insufficient ATP supply and ultimately repressing myoblast differentiation. This may also be the reason of the decrease in the number and diameter of muscle fibers in the SLD chicken skeletal muscle. Interestingly, previous studies have found that overexpression of *GHR* inhibits the formation of myotubes and the expression of myoblast differentiation markers [10]. We also revealed that overexpression of *GHR* inhibited myoblast differentiation, which contradicts with the result of *GHR* overexpression promoted mitochondrial function during myoblast differentiation. This may be because the mRNA expression has reached at an exceeded level compared to the normal physiological conditions after *GHR* overexpression. Excessive accumulation of *GHR* mRNA may activate a certain feedback regulation mechanism to inhibit the myoblast differentiation. Or excessive ROS production, induced by enhanced mitochondrial function after *GHR* overexpression, may inhibit the process of myoblast differentiation.

IGF1 combines with IGF1R to activate a variety of downstream pathways to regulate cell activity. Thus, we try to explore the signal transduction involved in the regulation of mitochondrial biogenesis by *IGF1*; thereby unveil the mechanism by which *GHR* regulates mitochondrial biogenesis through *IGF1*. AKT overexpression enhances myoblast differentiation [63]. Phosphorylated AKT is decreased in the whole tissue homogenate of GHRKO mice, while is increased in bovine GH transgenic mice [64]. The protein levels of p-PI3K and p-AKT are decreased after knocking down *GHR* in gastric cancer cell lines [65]. This is consistent with the result that *GHR* promotes the protein level of p-AKT during myoblast differentiation. CREB is a transcription factor that induces the transcription of more than 100 genes under the control of cAMP response elements, including *PGC1α* [40]. Several studies have shown that AKT can activate CREB activity and control cells survival [39, 66–68]. Knockdown of *IGF1* in chicken cardiomyocytes and myoblasts reduce the protein level of p-AKT [69, 70]. Inhibition of the PI3K signaling pathway reduces the quality of mitochondria and the expression of OXPHOS-related genes, while inhibition of the MAPK signaling pathway has no effect on the quality of mitochondria [22, 71]. Similarly, we revealed that *IGF1* promoted the protein level of p-AKT and p-CREB during myoblast differentiation; inhibitors of PI3K/AKT signaling pathway significantly down-regulated the expression of *PGC1α* and its protein level. These compelling evidences demonstrate that *IGF1* regulates mitochondrial biogenesis through the PI3K/AKT signaling pathway.

In conclusion, we corroborate that local *GHR* acts as a control valve to enhance mitochondrial function by promoting mitochondrial biogenesis via IGF1-PI3K/AKT/CREB pathway during myoblast differentiation.

## Supplementary Information

**Additional file 1: Table S1. **The sequences of primers for qRT-PCR. **Table S2. **The sequences of siRNA for *GHR* and *IGF1.*

**Additional file 2: Fig. S1 **High *GHR *expression during myoblast differentiation. a The *GHR* expression profile in the CPM proliferation and differentiation phases. b Transfection of overexpression plasmid or siRNA during CPM differentiation. c Overexpression efficiency of plasmid was measured by EGFP fluorescence intensity at 48 h after transfection with pcDNA3.1-EGFP during the CPM differentiation; scaler bar, 200 μm. d Knockdown efficiency of siRNA was measured by Cy3 fluorescence intensity at 48 h after transfection with siNC-Cy3 during the CPM differentiation; scaler bar, 200 μm. Data are shown as mean ± SEM, **p < 0.01.** Fig. S2 ***GHR* overexpression promotes mitochondrial biogenesis. a Overexpression efficiency was measured by RT-qPCR at 48 h after transfection with pcDNA3.1-*GHR *and pcDNA3.1. b The expression of genes involved in the GH-GHR-IGFs signaling pathway was measured by RT-qPCR at 48 h after transfection with pcDNA3.1-*GHR* and pcDNA3.1. c and d MTG staining of CPM was measured at 48 h after transfection with pcDNA3.1-*GHR* and pcDNA3.1. White arrow labeled elongated myoblasts. Scaler bar, 25 μm. e and f Confocal images were observed at 48 h after co-transfection with p*MitoTimer* + pcDNA3.1-*GHR* and p*MitoTimer* + pcDNA3.1-*GHR*. Scaler bar, 10 μm. Green represents newly synthesized mitochondria, red represents mature mitochondria. Images were analyzed by Leica LAS X life science software. g The expression of genes involved in PGC1α-NRF1-TFAM signaling pathway was measured by RT-qPCR at 48 h after transfection with pcDNA3.1-*GHR* and pcDNA3.1. h The expression of mtDNA encoded genes was measured by RT-qPCR at 48 h after transfection with pcDNA3.1-*GHR* and pcDNA3.1. i The relative mtDNA content was measured by RT-qPCR at 48 h after transfection with pcDNA3.1-*GHR* and pcDNA3.1. j-l Western blots with anti-GHR, anti-PGC1α, anti-NRF1 and anti-β-actin at48 h after transfection with pcDNA3.1-*GHR* and pcDNA3.1. Data are shownas mean ± SEM, *p < 0.05, **p< 0.01.** Fig. S3 ***IGF1* overexpression promotes mitochondrial biogenesis. a Overexpression efficiency was measured by RT-qPCR at 48 h after transfection with pcDNA3.1-*IGF1*and pcDNA3.1. b The expression of genes involved in the GH-GHR-IGFs signaling pathway was measured by RT-qPCR at 48 h after transfection with pcDNA3.1-*IGF1* and pcDNA3.1. c and d MTG staining of CPM was measured at 48 h after transfection with pcDNA3.1-*IGF1* and pcDNA3.1. White arrow labeled elongated myoblasts. Scaler bar, 25 μm. e and f Confocal images were observed at 48 h after co-transfection with p*MitoTimer *+ pcDNA3.1-*IGF1* and p*MitoTimer* + pcDNA3.1-*IGF1*. Scaler bar, 10 μm. Green represents newly synthesized mitochondria, red represents mature mitochondria. Images were analyzed by Leica LAS X life science software. g The expression of genes involved in PGC1α-NRF1-TFAM signaling pathway was measured by RT-qPCR at 48 h after transfection with pcDNA3.1-*IGF1 *and pcDNA3.1. h The expression of mtDNA encoded genes was measured by RT-qPCR at 48 h after transfection with pcDNA3.1-*IGF1* and pcDNA3.1. i The relative mtDNA content was measured by RT-qPCR at 48 h after transfection with pcDNA3.1-*IGF1* and pcDNA3.1. j and k Western blots with anti-PGC1α, anti-NRF1 and anti-β-actin at 48 h after transfection with pcDNA3.1-*IGF1* and pcDNA3.1. Data are shown as mean ± SEM, *p < 0.05, **p** < **0.01.** Fig. S4 **Local* GHR* regulates mitochondrial biogenesis via IGF1-PI3K/AKT/CREB signaling. a and b Western blots with anti-JAK2, anti-p-JAK2, anti-AKT1, anti-p-AKT1, anti-CREB1, anti-p-CREB1 andanti-β-actin at 48 h after transfection with pcDNA3.1-*GHR* and pcDNA3.1. c and d Western blots with anti-AKT1, anti-p-AKT1, anti-CREB1, anti-p-CREB1 and anti-β-actin at 48 h after transfection with pcDNA3.1-*IGF1* and pcDNA3.1. e The expression of *CREB* and *PGC1α* was measured by RT-qPCR at 48 h after transfection with si-*CREB *and si-NC. f and g Western blots with anti-PGC1α, anti-CREB1, anti-p-CREB1 and anti-β-actin at 48 h after transfection with si-*CREB *and si-NC. h Dual-Luciferase report assays of *CREB *knockdown co-transfected with reporter vectors containing different length of 5′ upstream region of *PGC1α*. Data are shown as mean ± SEM, *p < 0.05, **p < 0.01.** Fig. S5** *GHR* or *IGF1* overexpression enhances mitochondrial function. a ΔΨm was measured by the fluorescence of JC-1 at 48 h after transfection with pcDNA3.1-*GHR* and pcDNA3.1. b ΔΨm was measured by the fluorescence of JC-1 at 48 h after transfectionwith pcDNA3.1-*IGF1* and pcDNA3.1. c ATP level was measured at 48 h after transfection with pcDNA3.1-*GHR* and pcDNA3.1. d Reactive oxygen species production was measured by the fluorescence of DCF at 48 h after transfection with pcDNA3.1-*GHR* and pcDNA3.1. e ATP level was measured at 48 h after transfection with pcDNA3.1-*IGF1 *and pcDNA3.1. f Reactive oxygen species production was measured by the fluorescence of DCF at 48 h after transfection with pcDNA3.1-*IGF1 *and pcDNA3.1. Data are shown as mean ± SEM, *p < 0.05, **p< 0.01.** Fig. S6 ***GHR* overexpression represses myoblast differentiation. a CCK-8 assays were performed after transfection with pcDNA3.1-*GHR *and pcDNA3.1. b and c EdU proliferation assays were performed after transfection with pcDNA3.1-*GHR* and pcDNA3.1. d Cell cycle analysis were performed after transfection with pcDNA3.1-*GHR *and pcDNA3.1. e The expression of cell proliferation marker genes was measured by RT-qPCR at 48 h after transfection with pcDNA3.1-*GHR *and pcDNA3.1. f-h MyHC staining, myotube area and myoblast fusion index were measured at 48 h after transfection with pcDNA3.1-*GHR* and pcDNA3.1. i The expression of myoblast differentiation marker genes was measured by RT-qPCR at 48 h after transfection with pcDNA3.1-*GHR *and pcDNA3.1. Data are shown as mean ± SEM, *p < 0.05, **p < 0.01.

## Data Availability

All data generated or analyzed during this study are available from the corresponding author on reasonable request.

## Abbreviations

ATP: Adenosine triphosphate
cGH: Chicken GH
CPM: Chicken primary myoblast
CCK-8: Cell Counting Kit-8
DM: Differentiation medium
EdU: 5-Ethynyl-20-deoxyuridine
GHR: Growth hormone receptor
GM: Growth medium
IGF1: Insulin-like growth factor 1
IGFBP3: IGF-binding protein 3
JAKs: Janus kinases
MAPK: Mitogen-activated protein kinases
MTG: Mito-Tracker Green
mtDNA: Mitochondrial DNA
ΔΨm: Mitochondrial membrane potential
nDNA: Nuclear DNA
OXPHOS: Oxidative phosphorylation
PI3K: Phosphoinositide-3-kinase
PKB: Protein kinase B
PLC: Phospholipase C
PKC: Protein kinase C
PCR: Polymerase chain reaction
RT-qPCR: Real-time quantitative PCR
ROS: Reactive oxygen species
RIPA: Radio immune precipitation assay
STATs: Signal transducers and activators of transcription
SLD: Sex-linked dwarf
