# Supplementary material for: Local GHR roles in regulation of mitochondrial function through mitochondrial biogenesis during myoblast differentiation
Source: Cell Commun Signal. 2023 Jun 19;21:148. doi: 10.1186/s12964-023-01166-5 (PMC10278349; doi:10.1186/s12964-023-01166-5)
Supplement: Supplementary file 2 — Additional file 1: Table S1. The sequences of primers for qRT-PCR. Table S2. The sequences of siRNA for GHR and IGF1. [file 12964_2023_1166_MOESM1_ESM.docx]

**Tables**

**Table S1.** The sequences of primers for qRT-PCR.

**Table S2.** The sequences of siRNA for *GHR* and *IGF1*

**Table S1.** The sequences of primers for qRT-PCR.

| Gene | Primer sequence, 5’ to 3’ | Size, bp | Notes |
| --- | --- | --- | --- |
| *GH* | F-TGCAATACCTAAGCAAGGTGTTCA  R-CAGGGCTTGGATCCCTTCTT | 101 | qRT-PCR |
| *GHR* | F-GCGTGTTCAGGAGCAAAGCT  R-TGGGACAGGCATTTCCATACTT | 121 | qRT-PCR |
| *IGF1* | F-AGTTCGTATGTGGAGACAGAGGC  R-CCAGCCTCCTCAGGTCACAAC | 129 | qRT-PCR |
| *IGF2* | F-AGACCAGTGGGACGAAATAACA  R-CACGCTCTGACTTGACGGAC | 121 | qRT-PCR |
| *PGC1α* | F-ACTACGAACGATGACCCTCC  R-CCCTTGGGATCATTTGGAGACT | 173 | qRT-PCR |
| *PGC1β* | F-CTTGGCGCAAACCGACTCT  R-CTCCTCAGCAGGCTTGTACG | 121 | qRT-PCR |
| *PRC* | F-AAGGAACGTGCAATAGAGGAGC  R-GCGATAGGTGACAAAGCCGTA | 165 | qRT-PCR |
| *NRF1* | F-ACGAGGACTCACCTTCCTCA  R-TGTGGTCGCTTCCGTTTCTT | 163 | qRT-PCR |
| *NRF2* | F-TAGATGACAATGAGGCTTCTCC  R-CACTGTAACTCTGGAAGGGACA | 196 | qRT-PCR |
| *TFAM* | F-GACCTCGAAGTGGCTTCAAC  R-GAGCAAGCTGAAGGTATGGCT | 144 | qRT-PCR |
| *ND1* | F-ACCCAAGAGCCCATCTACCT  R-GTCCGGCGGCATATTCTACA | 154 | qPCR, qRT-PCR |
| *CYTB* | F-CAGCAGACACATCCCTAGCC  R-GAAGAATGAGGCGCCGTTTG | 104 | qRT-PCR |
| *COX1* | F-ACTACTTACCGACCGCAACC  R-CCGAAACCTGGGAGGATGAG | 132 | qRT-PCR |
| *ATP6* | F-TACAGCCACAATCGCCCTAC  R-AGGACGAAGACGTAGGCTTG | 123 | qRT-PCR |
| *tRNA-Leu* | F-GCTCGGCAAATGCAAAAGG  R-AGGATTTGAACCTCTGGATAAAGGG | 50 | qPCR |
| *MYOD* | F-GCTACTACACGGAATCACCAAAT  R-CTGGGCTCCACTGTCACTCA | 200 | qRT-PCR |
| *MYOG* | F-CGGAGGCTGAAGAAGGTGAA  R-CGGTCCTCTGCCTGGTCAT | 320 | qRT-PCR |
| *MYHC* | F-CTCCTCACGCTTTGGTAA  R-TGATAGTCGTATGGGTTGGT | 213 | qRT-PCR |
| *NFATc2* | F-AGGCTGGTTCATTACCACCA  R-CTGGCTGTCCATATCTGCCC | 184 | qRT-PCR |
| *PCNA* | F-GTGCTGGGACCTGGGTT  R-CGTATCCGCATTGTCTTCT | 217 | qRT-PCR |
| *CDKN1B* | F-TCGCTGTGCTGGGCTGAA  R-CAAGGACGAAAGGATGTGGG | 212 | qRT-PCR |
| *β-actin* | F-GATATTGCTGCGCTCGTTG  R-TTCAGGGTCAGGATACCTCTTT | 178 | qRT-PCR |

**Table S2.** The sequences of siRNA for *GHR* and *IGF1.*

| Gene | siRNA sequence, 5’ to 3’ | Notes |
| --- | --- | --- |
| *GHR* | GCTGTAACGAGGACACTTA | si-*GHR* |
| *IGF1* | GGAAGTGCATTTGAAGAAT | si-*IGF1* |
| *CREB1* | GGAGTTGAAAGCACTTAAA | si-*CREB* |
