# Supplementary material for: Local GHR roles in regulation of mitochondrial function through mitochondrial biogenesis during myoblast differentiation
Source: Cell Commun Signal. 2023 Jun 19;21:148. doi: 10.1186/s12964-023-01166-5 (PMC10278349; doi:10.1186/s12964-023-01166-5)
Supplement: Supplementary file 3 — Additional file 2: Fig. S1 High GHR expression during myoblast differentiation. a The GHR expression profile in the CPM proliferation and differentiation phases. b Transfection of overexpression plasmid or siRNA during CPM differentiation. c Overexpression efficiency of plasmid was measured by EGFP fluorescence intensity at 48 h after transfection with pcDNA3.1-EGFP during the CPM differentiation; scaler bar, 200 μm. d Knockdown efficiency of siRNA was measured by Cy3 fluorescence intensity at 48 h after transfection with siNC-Cy3 during the CPM differentiation; scaler bar, 200 μm. Data are shown as mean ± SEM, **p < 0.01. Fig. S2 GHR overexpression promotes mitochondrial biogenesis. a Overexpression efficiency was measured by RT-qPCR at 48 h after transfection with pcDNA3.1-GHR and pcDNA3.1. b The expression of genes involved in the GH-GHR-IGFs signaling pathway was measured by RT-qPCR at 48 h after transfection with pcDNA3.1-GHR and pcDNA3.1. c and d MTG staining of CPM was measured at 48 h after transfection with pcDNA3.1-GHR and pcDNA3.1. White arrow labeled elongated myoblasts. Scaler bar, 25 μm. e and f Confocal images were observed at 48 h after co-transfection with pMitoTimer + pcDNA3.1-GHR and pMitoTimer + pcDNA3.1-GHR. Scaler bar, 10 μm. Green represents newly synthesized mitochondria, red represents mature mitochondria. Images were analyzed by Leica LAS X life science software. g The expression of genes involved in PGC1α-NRF1-TFAM signaling pathway was measured by RT-qPCR at 48 h after transfection with pcDNA3.1-GHR and pcDNA3.1. h The expression of mtDNA encoded genes was measured by RT-qPCR at 48 h after transfection with pcDNA3.1-GHR and pcDNA3.1. i The relative mtDNA content was measured by RT-qPCR at 48 h after transfection with pcDNA3.1-GHR and pcDNA3.1. j-l Western blots with anti-GHR, anti-PGC1α, anti-NRF1 and anti-β-actin at48 h after transfection with pcDNA3.1-GHR and pcDNA3.1. Data are shownas mean ± SEM, *p < 0.05, **p< 0.01. Fig. S3 IGF1 over [file 12964_2023_1166_MOESM2_ESM.docx]

**Fig S1. High *GHR* expression during myoblast differentiation.** **a** The *GHR* expression profile in the CPM proliferation and differentiation phases. **b** Transfection of overexpression plasmid or siRNA during CPM differentiation. **c** Overexpression efficiency of plasmid was measured by EGFP fluorescence intensity at 48 h after transfection with pcDNA3.1-EGFP during the CPM differentiation; scaler bar, 200 μm. **d** Knockdown efficiency of siRNA was measured by Cy3 fluorescence intensity at 48 h after transfection with siNC-Cy3 during the CPM differentiation; scaler bar, 200 μm. Data are shown as mean ± SEM, ***p* < 0.01.

**Fig S2. *GHR* overexpression promotes mitochondrial biogenesis. a** Overexpression [efficiency](file:///C:\Users\%E5%95%8A%E6%B3%A2%E6%AC%A1%E7%9A%84\AppData\Local\youdao\dict\Application\7.5.2.0\resultui\dict\?keyword=efficiency) was measured by RT-qPCR at 48 h after transfection with pcDNA3.1-*GHR* and pcDNA3.1. **b** The expression of genes involved in the GH-GHR-IGFs signaling pathway was measured by RT-qPCR at 48 h after transfection with pcDNA3.1-*GHR* and pcDNA3.1. **c** and **d** MTG staining of CPM was measured at 48 h after transfection with pcDNA3.1-*GHR* and pcDNA3.1. White arrow labeled elongated myoblasts. Scaler bar, 25 μm. **e** and **f** Confocal images were observed at 48 h after co-transfection with p*MitoTimer* + pcDNA3.1-*GHR* and p*MitoTimer* + pcDNA3.1-*GHR*. Scaler bar, 10 μm. Green represents newly synthesized mitochondria, red represents mature mitochondria. Images were analyzed by Leica LAS X life science software. **g** The expression of genes involved in PGC1α-NRF1-TFAM signaling pathway was measured by RT-qPCR at 48 h after transfection with pcDNA3.1-*GHR* and pcDNA3.1. **h** The expression of mtDNA encoded genes was measured by RT-qPCR at 48 h after transfection with pcDNA3.1-*GHR* and pcDNA3.1. **i** The relative mtDNA content was measured by RT-qPCR at 48 h after transfection with pcDNA3.1-*GHR* and pcDNA3.1. **j-l** Western blots with anti-GHR, anti-PGC1α, anti-NRF1 and anti-β-actin at 48 h after transfection with pcDNA3.1-*GHR* and pcDNA3.1. Data are shown as mean ± SEM, **p* < 0.05, ***p* < 0.01.

**Fig S3. *IGF1* overexpression promotes mitochondrial biogenesis.** **a** Overexpression [efficiency](file:///C:\Users\%E5%95%8A%E6%B3%A2%E6%AC%A1%E7%9A%84\AppData\Local\youdao\dict\Application\7.5.2.0\resultui\dict\?keyword=efficiency) was measured by RT-qPCR at 48 h after transfection with pcDNA3.1-*IGF1* and pcDNA3.1. **b** The expression of genes involved in the GH-GHR-IGFs signaling pathway was measured by RT-qPCR at 48 h after transfection with pcDNA3.1-*IGF1* and pcDNA3.1. **c** and **d** MTG staining of CPM was measured at 48 h after transfection with pcDNA3.1-*IGF1* and pcDNA3.1. White arrow labeled elongated myoblasts. Scaler bar, 25 μm. **e** and **f** Confocal images were observed at 48 h after co-transfection with p*MitoTimer* + pcDNA3.1-*IGF1* and p*MitoTimer* + pcDNA3.1-*IGF1*. Scaler bar, 10 μm. Green represents newly synthesized mitochondria, red represents mature mitochondria. Images were analyzed by Leica LAS X life science software. **g** The expression of genes involved in PGC1α-NRF1-TFAM signaling pathway was measured by RT-qPCR at 48 h after transfection with pcDNA3.1-*IGF1* and pcDNA3.1. **h** The expression of mtDNA encoded genes was measured by RT-qPCR at 48 h after transfection with pcDNA3.1-*IGF1* and pcDNA3.1. **i** The relative mtDNA content was measured by RT-qPCR at 48 h after transfection with pcDNA3.1-*IGF1* and pcDNA3.1. **j** and **k** Western blots with anti-PGC1α, anti-NRF1 and anti-β-actin at 48 h after transfection with pcDNA3.1-*IGF1* and pcDNA3.1. Data are shown as mean ± SEM, **p* < 0.05, ***p* < 0.01.

**Fig S4. Local *GHR* regulates mitochondrial biogenesis via IGF1-PI3K/AKT/CREB signaling.** **a** and **b** Western blots with anti-JAK2, anti-p-JAK2, anti-AKT1, anti-p-AKT1, anti-CREB1, anti-p- CREB1 and anti-β-actin at 48 h after transfection with pcDNA3.1-*GHR* and pcDNA3.1. **c** and **d** Western blots with anti-AKT1, anti-p-AKT1, anti-CREB1, anti-p-CREB1 and anti-β-actin at 48 h after transfection with pcDNA3.1-*IGF1* and pcDNA3.1. **e** The expression of *CREB* and *PGC1α* was measured by RT-qPCR at 48 h after transfection with si-*CREB* and si-NC. **f** and **g** Western blots with anti-PGC1α, anti-CREB1, anti-p-CREB1 and anti-β-actin at 48 h after transfection with si-*CREB* and si-NC. **h** Dual-Luciferase report assays of *CREB* knockdown co-transfected with reporter vectors containing different length of 5′ upstream region of *PGC1α***.** Data are shown as mean ± SEM, **p* < 0.05, ***p* < 0.01.

**Fig S5. *GHR* or *IGF1* overexpression enhances mitochondrial function.**  **a** ΔΨm was measured by the fluorescence of JC-1 at 48 h after transfection with pcDNA3.1-*GHR* and pcDNA3.1. **b** ΔΨm was measured by the fluorescence of JC-1 at 48 h after transfection with pcDNA3.1-*IGF1* and pcDNA3.1. **c** ATP level was measured at 48 h after transfection with pcDNA3.1-*GHR* and pcDNA3.1. **d** Reactive oxygen species production was measured by the fluorescence of DCF at 48 h after transfection with pcDNA3.1-*GHR* and pcDNA3.1. **e** ATP level was measured at 48 h after transfection with pcDNA3.1-*IGF1* and pcDNA3.1. **f** Reactive oxygen species production was measured by the fluorescence of DCF at 48 h after transfection with pcDNA3.1-*IGF1* and pcDNA3.1 (shared control group with pcDNA3.1-*GHR*). Data are shown as mean ± SEM, **p* < 0.05, ***p* < 0.01.

**Fig S6. *GHR* overexpression represses myoblast differentiation. a** CCK-8 assays were performed after transfection with pcDNA3.1-*GHR* and pcDNA3.1. **b** and **c** EdU proliferation assays were performed after transfection with pcDNA3.1-*GHR* and pcDNA3.1. **d** Cell cycle analysis were performed after transfection with pcDNA3.1-*GHR* and pcDNA3.1. **e** The expression of cell proliferation marker genes was measured by RT-qPCR at 48 h after transfection with pcDNA3.1-*GHR* and pcDNA3.1. **f-h** MyHC staining, myotube area and myoblast fusion index were measured at 48 h after transfection with pcDNA3.1-*GHR* and pcDNA3.1. **i** The expression of myoblast differentiation marker genes was measured by RT-qPCR at 48 h after transfection with pcDNA3.1-*GHR* and pcDNA3.1. Data are shown as mean ± SEM, **p* < 0.05, ***p* < 0.01.
